# Targeting de novo loss-of-function variants in constrained disease genes improves diagnostic rates in the 100,000 Genomes Project

**DOI:** 10.1007/s00439-022-02509-x

**Published:** 2022-12-07

**Authors:** Eleanor G. Seaby, N. Simon Thomas, Amy Webb, Helen Brittain, Ana Lisa Taylor Tavares, Genomics England Consortium, J. C. Ambrose, P Arumugam, R Bevers, M Bleda, F Boardman-Pretty, C. R. Boustred, H Brittain, M. J. Caulfield, G. C. Chan, T Fowler, A Giess, A Hamblin, S Henderson, T. J. P. Hubbard, R Jackson, L. J. Jones, D Kasperaviciute, M Kayikci, A Kousathanas, L. Lahnstein, S. E. A. Leigh, I. U. S. Leong, F. J. Lopez, F Maleady-Crowe, M. McEntagart, F Minneci, L Moutsianas, M. Mueller, N Murugaesu, A. C. Need, P. O‘Donovan, C. A. Odhams, C Patch, D Perez-Gil, M. B. Pereira, J Pullinger, T Rahim, A Rendon, T Rogers, K Savage, K Sawant, R. H. Scott, A Siddiq, A Sieghart, S. C. Smith, A Sosinsky, A Stuckey, M Tanguy, A. L. Taylor Tavares, E. R. A. Thomas, S. R. Thompson, A Tucci, M. J. Welland, E Williams, K Witkowska, S. M. Wood, Diana Baralle, Heidi L. Rehm, Anne O’Donnell-Luria, Sarah Ennis

**Affiliations:** 1 Genomic Informatics Group, Human Development and Health, Faculty of Medicine, University Hospital Southampton, MP 808, Duthie Building, Southampton, SO16 6YD Hampshire UK; 2 Program in Medical and Population Genetics, Broad Institute of MIT and Harvard, Cambridge, MA 02142 USA; 3 Division of Genetics and Genomics, Boston Children’s Hospital, Boston, MA 02115 USA; 4 Paediatric Infectious Diseases, Imperial College London, London, W2 1NY UK; 5 Wessex Regional Genomics Laboratory, Salisbury NHS Foundation Trust, Salisbury, SP2 8BJ UK; 6 Genomics England, Charterhouse Square, London, EC1M 6BQ UK; 7 East Anglian Medical Genetics Service, Cambridge University Hospital, Hills Road, Cambridge, CB2 0QQ UK; 8 Center for Genomic Medicine, Massachusetts General Hospital, Boston, MA 02114 USA

## Abstract

**Background:**

Genome sequencing was first offered clinically in the UK through the 100,000 Genomes Project (100KGP). Analysis was restricted to predefined gene panels associated with the patient’s phenotype. However, panels rely on clearly characterised phenotypes and risk missing diagnoses outside of the panel(s) applied. We propose a complementary method to rapidly identify pathogenic variants, including those missed by 100KGP methods.

**Methods:**

The Loss-of-function Observed/Expected Upper-bound Fraction (LOEUF) score quantifies gene constraint, with low scores correlated with haploinsufficiency. We applied DeNovoLOEUF, a filtering strategy to sequencing data from 13,949 rare disease trios in the 100KGP, by filtering for rare, de novo, loss-of-function variants in disease genes with a LOEUF score < 0.2. We compared our findings with the corresponding patient’s diagnostic reports.

**Results:**

324/332 (98%) of the variants identified using DeNovoLOEUF were diagnostic or partially diagnostic (whereby the variant was responsible for some of the phenotype). We identified 39 diagnoses that were “missed” by 100KGP standard analyses, which are now being returned to patients.

**Conclusion:**

We have demonstrated a highly specific and rapid method with a 98% positive predictive value that has good concordance with standard analysis, low false-positive rate, and can identify additional diagnoses. Globally, as more patients are being offered genome sequencing, we anticipate that DeNovoLOEUF will rapidly identify new diagnoses and facilitate iterative analyses when new disease genes are discovered.

**Supplementary Information:**

The online version contains supplementary material available at 10.1007/s00439-022-02509-x.

## Introduction

With transformative advances in genomic medicine, there has been an exponential rise in the number of individuals undergoing exome and genome sequencing. A shift towards large-scale international sequencing programs is improving affordability and accessibility of such sequencing for diagnostic purposes, where conventional clinical tests have failed to yield a diagnosis (Seaby et al. 2021; Posey et al. 2019). The 100,000 Genomes Project (100KGP) was a research project embedded within the UK National Health Service and the precursor to offering whole genome sequencing (WGS) as a clinical test (Turnbull et al. 2018; Parliament 2015; The 100,000 Genomes Project Pilot Investigators 2021). This pioneering project benefited from sequencing vast patient numbers with rare genetic diseases with improved power to identify multiple patients with overlapping phenotypes and genotypes; however, the number of cases that required clinical assessment for diagnostic reporting versus resources available created a significant bottleneck.

**Fig. 1:**
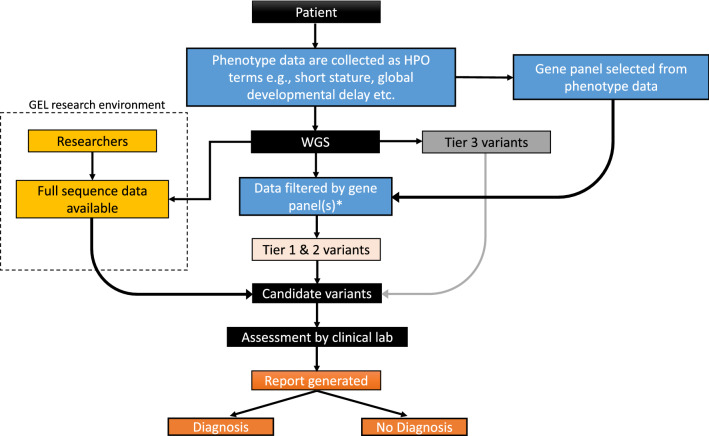
Genomics England 100,000 Genomes Project workflow. Phenotype data were collected from patients and recorded as HPO terms. These terms informed the virtual gene panel(s) applied for data analysis. This contrasts with the new UK Genome Medicine Service, where the clinician selects the panel(s) applied. In 100KGP, the patient underwent whole genome sequencing (WGS), and their sequencing data were filtered using the pre-selected gene panel(s). Data were also filtered by allele frequency and variant segregation (*) and these variants were classified into Tier 1 and Tier 2 variants as previously described (The 100,000 Genomes Project Pilot Investigators 2021). Candidate variants within the gene panel were identified and assessed by an NHS accredited diagnostic laboratory, and a report was generated and returned to the patient. Diagnostic laboratories were under no obligation to review variants outside of the virtual panel(s) applied; however, rare variants outside of the predefined panel(s) were available for analysis as Tier 3 variants. Variants outside of the gene panel(s), including full raw sequencing data, remain accessible to approved researchers for interrogation as fully anonymised data. Potential candidate variants identified through this route can be reported via Genomics England for potential return for local review by clinical laboratories

Diagnostic rates for the 100KGP were similar to the international average for rare diseases (Rehm 2022). The flagship 100KGP paper showed that an estimated diagnostic uplift from 15 to 20% could be achieved beyond prior testing, but that the time and level of additional resources required to analyse the full genome was beyond routine diagnostic testing (The 100,000 Genomes Project Pilot Investigators 2021). As a result, the project adopted the use of predefined gene panels (Genomics England PanelApp) (Martin et al. 2019) to target sequencing analysis to the most relevant genes selected from the Human Phenotype Ontology (HPO) (Robinson et al. 2008) terms provided by the referring clinician (Fig. 1) (The 100,000 Genomes Project Pilot Investigators 2021). Whilst this approach restricted the number of variants assessed and improved the efficiency of the variant curation process applied to each patient’s genome for diagnostic reporting, it risked missing variants in genes outside of the gene panel applied. At the latter stages of the project, many NHS England clinical laboratories additionally reviewed all de novo variants and top Exomiser (Smedley et al. 2015) results, although this was never mandatory. This learning has also informed guidance for the evaluation of genome sequencing through the new Genome Medicine Service in the UK.

Accurate phenotyping is essential for gene panel selection, yet there is huge variability in the phenotypes reported by clinicians. For some cases in the 100KGP, only a single HPO term was reported. As WGS becomes more widespread, appropriate matching of HPO terms with optimal panel(s) may be less error prone for experienced geneticists but will represent a challenge for the wider community of clinicians expected to routinely refer patients. Furthermore, whilst the use of HPO terms aids in the standardization of reporting phenotype data, it represents a cross-sectional time-point analysis without resource for re-analysis. Ultimately, using HPO terms lacks the full clinical narrative and challenges gene panel selection. Therefore, there is need to expand genome analysis beyond gene panels to enable a more agnostic and comprehensive genome analysis, yet this needs to be balanced with the number of variants that require manual assessment for diagnostic reporting. Targeting variants with high pathogenic potential across the entire exome provides an opportunity to rapidly identify diagnostic variants and uplift diagnostic rates. Genotype-driven analysis approaches are complementary to the phenotype-drive approach currently utilized by 100KGP.

**Fig. 2:**
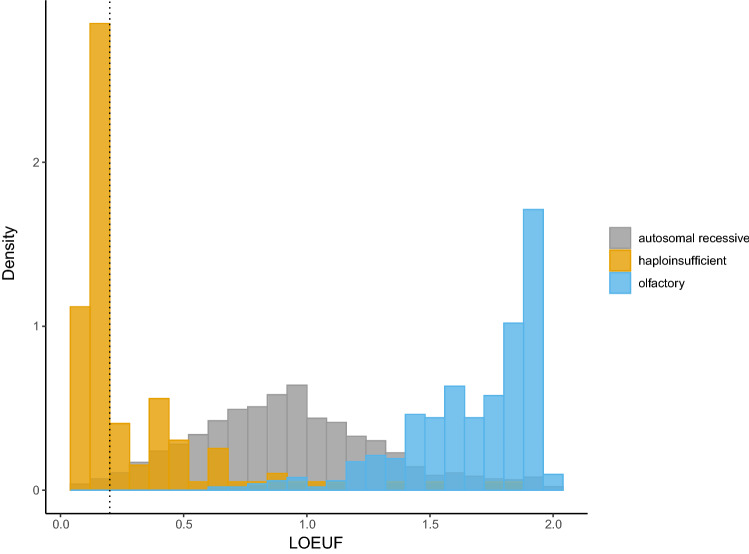
Density histogram of the LOEUF score compared with manually curated haploinsufficient, autosomal recessive, and olfactory gene lists using data publicly available from: https://github.com/broadinstitute/gnomad_lof. Haploinsufficient genes are enriched for low LOEUF scores, with a natural cut off at 0.2 (dotted line). Autosomal recessive genes sit in the middle of the distribution and olfactory genes show tolerance to loss-of-function with high LOEUF scores. Inspiration for figure taken by Karczewski et al. with permission (Karczewski et al. 2020)

Looking at variation across people, some genes are extremely depleted or constrained for variation predicted to result in loss-of-function (LoF) (MacArthur et al. 2012). That is to say, there is negative selection against the loss or inactivation of one allele. By comparing the observed over the expected rate of predicted loss-of-function variants in large population databases, it is now possible to compute the degree of constraint a given gene has for inactivation (Gudmundsson et al. 2021; Karczewski et al. 2020). The loss-of-function observed over expected upper bound fraction, or LOEUF score, is a metric that places each gene on a continuous scale of loss-of-function constraint. Low scores are highly correlated with disease genes and gene essentiality, with the first LOEUF decile (< 0.2) being enriched for haploinsufficient disease genes (Fig. 2) and the greatest burden of OMIM disease entries (Karczewski et al. 2020). Loss-of-function variants in extremely LoF constrained genes are therefore prime targets for potential diagnoses.

We aimed to utilise the sequencing and phenotype data generated through 100KGP and apply a transferable and rapid filtering method, we called DeNovoLOEUF, that can screen for putative pathogenic variants. We developed a rapid, agnostic approach to target the highest diagnostic yield variants in rare disease patients whilst enabling clinical curators to focus on the most important findings, regardless of the gene panel applied, improving efficiency for cases where a diagnosis could be rapidly identified.

## Materials and methods

### Data access

We obtained access to the GEL research environment (RE) and high-performance cluster (HPC) behind a secure firewall following information governance training and with membership of a Genomics England Clinical Interpretation Partnership (GeCIP): *Quantitative methods, machine learning, and functional genomics*. We had a project approved (RR359—*Translational genomics: Optimising novel gene discovery for 100,000 rare disease patients*) which permitted access to anonymised 100KGP sequencing and phenotype data. This included an aggregate *vcf* file comprising 13,949 rare disease trios with de novo variants, called using the Illumina Platypus pipeline (The 100,000 Genomes Project Pilot Investigators 2021).

### Phenotype data

Referring clinicians recorded phenotype data as categorical HPO terms. These were accessible in the RE by querying HPO terms stored in mysql tables in a LabKey data management system. Gene panels were selected by GEL based on the phenotype terms provided. A summary of high-level phenotypes of the patient population is available in Supplementary Data 1.

### Data analysis

**Fig. 3:**
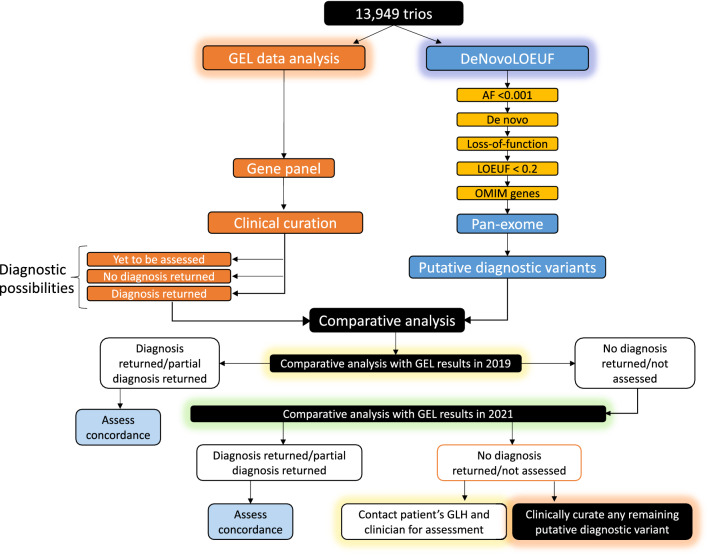
Summary of methods. Putative diagnostic variants identified by our filtering approach were compared with the diagnostic reports for the same patients. Following comparative analysis in 2021, if a negative report had been issued, or the case was still under review, we contacted the patient’s Genomics Laboratory Hub and referring clinician and shared our variant of interest. If we received no response, we clinically curated the remaining variants as per ACMG-AMP guidelines (Richards et al. 2015) in an NHS approved diagnostic laboratory. *AF* allele frequency, *GEL* Genomics England, *GLH* Genomics Laboratory Hub, *LOEUF* Loss-of-function Observed/Expected Upper-bound Fraction, *OMIM* Online Mendelian Inheritance in Man

Data analysis was performed in Autumn 2019 (Fig. 3). Bespoke scripts were developed to query the aggregate *vcf* file. We selected only variants that passed Illumina QC as previously described (The 100,000 Genomes Project Pilot Investigators 2021). We then applied a filtering strategy called DeNovoLOEUF: First, we selected de novo variants with an allele frequency < 0.001 in gnomAD v2.1.1 (all populations). We further restricted this list to predicted loss-of-function (pLoF) variants including nonsense, frameshift and essential (canonical ± 2 base-pairs) splice site variants. We imported LOEUF constraint gene scores, downloaded from the gnomAD browser (https://gnomad.broadinstitute.org), into the research environment. We then restricted rare, de novo, pLoF variants to genes with a LOEUF score of < 0.2 (*n* = 1044), approximately equivalent to the first LOEUF decile, representing a list of genes most highly constrained for loss-of-function and predicted to cause disease through haploinsufficiency as outlined in the flagship gnomAD paper and visualised in Fig. 2 (Karczewski et al. 2020). We retained only those LOEUF constrained genes with known disease gene associations in the OMIM (Amberger et al. 2015) database (*n* = 335) accessed and downloaded as a flat*.txt* file in October 2019. We excluded known autosomal recessive disease genes leaving 293 genes. Variants in novel disease genes represent further potential diagnoses but are beyond the scope of this disease gene focused assessment and have been published elsewhere (Seaby et al. 2022). We applied LOFTEE v1.0 (Karczewski et al. 2020) to flag variants as potential false positives but retained variants in the terminal exon. Variants remaining following DeNovoLOEUF filtering steps were considered *putative diagnostic variants*.

Clinical outcome data pertaining to diagnostic reports and individual specific phenotype information were extracted querying Labkey using the RLabKey package (v2.9.0) in R (v4.0.3). These phenotype data were computationally merged with filtered putative pathogenic variants for each patient. We further extracted the diagnostic report status for each patient, which included any returned pathogenic variants, by computationally querying the ‘GMC exit questionnaire’ table in LabKey.

### Comparative analysis

Putative diagnostic variants were compared with the diagnoses returned to patients recruited to 100KGP at two time points (October 2019 and April 2021) to assess concordance between our DeNovoLOEUF filtering method and the analysis strategy by GEL.

At the first time-point, putative diagnostic variants extracted using DeNovoLOEUF were compared against variants declared in the Genome Medicine Centre exit questionnaire of the RE as being returned to patients in their diagnostic report. This was to assess the positive predictive value (PPV) of the method. It was expected that many patients would not have had a diagnostic report returned in 2019, i.e., their case status was “yet to be assessed”. The comparative analysis was therefore repeated at the second time-point (18 months later in April 2021), to assess whether our method correctly predicted additional diagnoses determined over time as the proportion of closed 100KGP cases increased.

Cases that were not assessed or reported as negative (i.e., no diagnosis identified) by 2021 were re-curated by NHS Clinical Scientists to standardize curation of any novel diagnoses not originally detected through the 100KGP. This was achieved in two ways. First, we contacted the patient’s Genomic Laboratory Hub (GLH), previously known as the Genome Medicine Centre, and referring clinician to discuss the variant we had found and asked whether the variant was already known about and/or had been returned as a diagnosis. Communication with the GLH and referring clinicians often prompted local multidisciplinary team meetings followed by diagnostic laboratory confirmation of the variant. Second, for the variants that were unknown to the GLHs, or for which we received no response from the centres contacted, we worked with clinical scientists in the Wessex Regional Genetics Laboratory, an established GEL diagnostic reporting centre, who curated the remaining variants alongside the patients’ phenotypes as per the ACMG-AMP guidelines (Richards et al. 2015). We then determined how many of the remaining putative diagnostics variants would meet a partial or full diagnosis.

### Testing the method on non-trio data

In June 2022, we filtered for rare (AF < 0.001), pLoF variants, in OMIM disease genes with a LOEUF score < 0.2 in an additional 6101 families with complex family structures whereby de novo analysis was not possible. This filtering strategy mimicked the DeNovoLOEUF strategy, except for removing the de novo filter. We retained variants present in affected individuals only. As before, we compared any putative diagnostic variants with the patient’s GMC exit questionnaire.

### Iterative re-analysis

In August 2022, we repeated DeNovoLOEUF on newly discovered disease genes with a LOEUF score < 0.2, published between 2019 and 2022, that were classified as ‘definitive’ or ‘strong’ in GenCC (DiStefano et al. 2022) to assess possible diagnostic uplift. These variants were then curated by a clinician scientist in an NHS diagnostic laboratory.

## Results

**Fig. 4:**
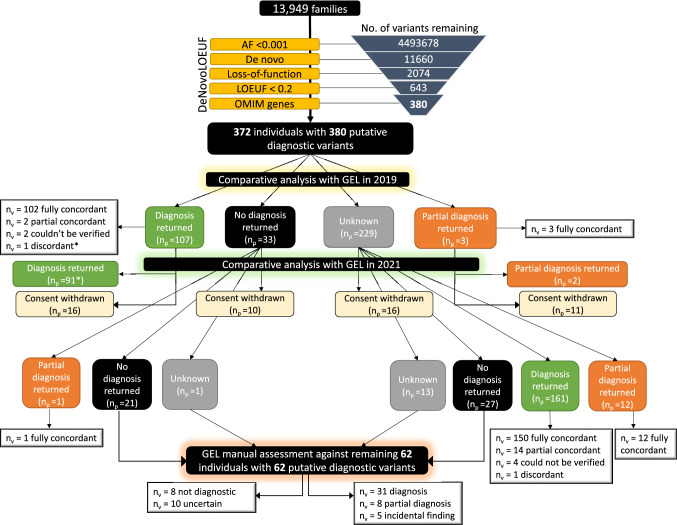
Summary of results using DeNovoLOEUF on 100,000 Genomes Project patients. In October 2019, 380 predicted loss-of-function variants in 372 individuals were identified in known OMIM disease genes using DeNovoLOEUF. At the time, 29% (107/372) of patients had a single diagnostic variant returned and 33 patients had their case closed as ‘negative’. Ninety-five percent (102/107) of variants identified by our method were entirely concordant with the formal GEL diagnosis returned. In two patients where we correctly identified a pathogenic variant, a second variant in a different gene also contributed to the diagnosis (partially concordant). Two variants were excluded from comparative analysis as it was not possible to verify the returned diagnosis in the patient's exit questionnaire. One variant was discordant (*) between the variant identified by DeNovoLOEUF and the reported outcome data from the 100KGP; however, this variant was subsequently confirmed as pathogenic by 2021. Three patients had a partial diagnosis returned, meaning a single variant was returned to the patient but that it did not fully explain the phenotype; all three were fully concordant with our method. 232 cases were ‘unknown’ meaning that no formal report had been returned to the patient. In summary, at the first time-point, the method correctly detected 99% (107/108) of reported diagnostic variants. For the comparative analysis in 2021, 43 variants were excluded from downstream analysis due to patients being withdrawn from 100KGP. A further 150 variants were fully concordant with our method, 14 were partially concordant, 13 variants were concordant with a returned partial diagnosis, and 1 variant was discordant. Following assessment of the remaining 65 variants in 65 individuals, 31 cases were considered diagnostic, 8 cases partially diagnostic, and 5 cases were incidental findings. Eight variants did not explain the phenotype, and 10 cases were uncertain, meaning that there was insufficient clinical information to determine causality. *n*_p_ = number of patients; *n*_v_ = number of variants

A total of 380 putative diagnostic variants were identified by DeNovoLOEUF in 372 patients. Of these variants, 339/380 (89%) were in the Exomiser top-ranked results. There were more variants than patients due to some individuals harbouring more than one de novo variant in the same gene (*n*_patients_ = 2) or having more than one de novo variant in two different genes (*n*_patients_ = 6). The patients with two de novo variants in the same gene were explored further and these variants did not represent a complex structural event. Results stratified by time-point assessment are shown in Fig. 4.

### Comparative analysis of method in 2021

In April 2021, 284/380 (75%) of all variants initially identified were confirmed as either fully diagnostic or partially diagnostic, including 17 patients who had diagnostic reports returned in 2019 prior to being withdrawn from the study. A single discordant variant identified in 2019 (Fig. 4) was reclassified as a pathogenic variant by 2021 and returned to the patient as diagnostic. Twenty-six patients harboring 26 variants identified in 2019 were unavailable for further assessment in 2021 due to either withdrawing from the 100KGP or data being temporarily removed from the trusted research environment, whilst re-consent was sought for child participants reaching adulthood. Only one variant that we identified did not match the variant returned to the patient, meaning GEL had returned an alternative diagnosis; however, the variant we identified is a known pathogenic variant in ClinVar. This patient had only one HPO term recorded “cystinosis”, which was consistent with the biallelic variants reported by GEL. We are attempting to contact the clinician responsible for this patient to gain further clinical information and establish whether the putative diagnostic variant identified may be a missed additional diagnosis. Six variants could not be assessed for concordance as the variant returned by 100KGP was not identifiable in the GEL research environment. Sixty-two variants in 62 individuals identified using DeNovoLOEUF remained unresolved in 2021 and required further scrutiny. All 62 variants were in Tier 3 of the GEL tiering system as they were de novo loss-of-function variants but were not in the original gene panel(s) applied.

### Assessment of remaining 62 variants in 62 unique individuals

We successfully established contact with 17 (27%) of the remaining 62 patient’s GLHs and referring clinicians. Following this connection, all 17 patients had their DeNovoLOEUF variants confirmed as disease-causing following independent validation in their local NHS laboratories. We were unable to establish contact with the remaining 45 patients’ referring clinician and/or GLH, and therefore, the outstanding 45 variants were manually curated by two clinical scientists working in an NHS accredited genomic diagnostic laboratory (Supplementary Data 2). Of these variants, 14 were designated diagnostic, 8 were partially diagnostic, and 5 were identified as incidental findings. Eight variants were considered not diagnostic, and ten cases were uncertain with insufficient clinical information to confirm causality for the patient’s phenotype (Table 1).

**Table 1:** Classification of remaining 62 variants

Diagnosis (*n* = 31)	Partial diagnosis (*n* = 8)	Not diagnostic (*n* = 8)	Uncertain (*n* = 10)	Incidental finding (*n* = 5)
Confirmed following GLH contact (*n* = 17)	Confirmed following manual curation (*n* = 8)	Poor phenotype fit (*n* = 3)	Pathogenic by ACMG-AMP guidelines (*n* = 5)	Confirmed following manual curation (*n* = 5)
Confirmed following manual curation (*n* = 14)		Intronic on MANE Select transcript (*n* = 2)	VUS by ACMG-AMP guidelines (*n* = 5)	
		XLR gene and patient female (*n* = 1)		
		Artefactual variant (*n* = 1)		
		Splice rescue (*n* = 1)		

Half (5/10) of the uncertain variants were classified as pathogenic by ACMG-AMP guidelines (Richards et al. 2015), but there was insufficient clinical information to confirm causality for the patient’s phenotype; there was an average of 4 HPO terms per ‘uncertain’ case compared with 18 HPO terms for patients with a diagnosis. Of the 8 non-diagnostic cases, one variant was in an X-linked recessive (XLR) gene and the patient was female. A further variant passed GEL quality control filtering in 2019 but upon more detailed inspection was artefactual. One variant was within six base-pairs of an alternative splice site, predicting a full splice rescue. Three variants were a poor phenotypic fit. Two variants were intronic on the Matched Annotation from NCBI and EBML-EBI (MANE) (Morales et al. 2022). Select transcript and not present in an exon of a MANE Plus Clinical alternative transcript harboring known pathogenic variants

### Summary of results

In summary, 324/332 (98%) of the variants identified through DeNovoLOEUF filtering (excluding incidental findings, variants for withdrawn participants, or in patients where there was inadequate phenotype or genotype reporting in GEL) were classified as diagnostic or partially diagnostic (Table 2).

**Table 2:** Tabulated summary of results

GEL diagnoses returned	2019 Comparison	2021 Comparison	Following curation/GLH
Fully concordant	102	253	284
Partially concordant	5	32	40
Could not be verified	2	6	16
Discordant	1 [discarded in 2021]	1	8
Incidental	0	0	5
PPV	107/108 = 99%	285/286 = 99%	324/332 = 98%

Summary of results comparing variants identified by our method compared with diagnostic reports returned to patients, in addition to variant curation. The 2021 comparison is a cumulative comparison of the 2021 and 2019 comparisons. Full concordance is where a single variant we identified was confirmed as the pathogenic variant. Partial concordance denotes the case where the variant we identified was pathogenic but did not explain the full phenotype, or a second variant in a different gene was returned to the patient. Variants that could not be verified were those where it was not possible to see which variant had been returned to the patient as diagnostic, or there was not enough clinical information to determine causality. These 16 variants, plus the 5 incidental findings were not included in the PPV calculations

*GLH* Genomics Laboratory Hub, *PPV* positive predictive value

### DeNovoLOEUF on non-trio data

Filtering for rare, pLoF variants in known OMIM disease genes with a LOEUF score < 0.2 on 6101 families with complex family structures revealed a further 776 putative diagnostic variants in 757 individuals. 270/757 (36%) of individuals had diagnoses returned that were fully concordant with our method. The number of individuals per pedigree was significantly different between concordant and discordant cases (Wilcoxon test, *p* = 6.02^–16^), with discordant patients having a median pedigree structure of one individual (singleton).

### Proportion of de novo disease-causing variants detected by DeNovoLOEUF

We sought to explore the total number of de novo pLoF variants detected by GEL and how many were captured by DeNovoLOEUF. A total of 2074 de novo pLoF were identified in 100KGP (Fig. 4). Of these, 480/2074 (23%) were confirmed diagnoses. Of these diagnoses, 380/480 (79.2%) were in LOEUF constrained genes (with a score < 0.2). We further expanded our method to test different cut offs, including a LOEUF score between 0.2 and 0.4 and between 0.4 and 0.6, whereby the positive predictive values were 69% and 45%, respectively.

### Iterative re-analysis

Re-running LOEUF on newly discovered genes between 2019 and 2022 that were not present in our original OMIM gene list identified 13 new de novo pLoF variants. 12/13 (92%) have been confirmed as diagnostic and are being returned to patients (Supplementary Data 3).

## Discussion

We describe a fast, unbiased filtering strategy, DeNovoLOEUF, to identify potential pathogenic variants with high positive predictive value and specificity. The LOEUF scores for genes are available in Supplementary Data 4. We utilise the LOEUF spectrum of constraint to identify de novo loss-of-function variants with a high potential of pathogenicity. Unlike the approach adopted in the 100KGP with panel-based tiering, our genotype-driven method is agnostic to phenotype and independent of gene panels which often change over time and require the correct one to be selected. Indeed, we identified 39 diagnostic or partially diagnostic variants in 39 known disease-associated genes that had been missed by standard 100KGP diagnostic protocols due to the gene in which the causal variant was identified not being included on the gene panel selected. 35/39 (90%) of these genes were included ‘green’ on different gene panels, meaning that they were recognised “Panel App disease genes”, but the gene panel selection was suboptimal. One of the issues with PanelApp is in selecting the ‘correct’ panel based on the HPO terms provided. For example, the gene *CLCN5* is a disease gene for Dent disease 1 (MIM: 300009), a recognised renal tubulopathy. This gene is on the following PanelApp gene panels: “nephrocalcinosis”, “unexplained kidney failure in young people”, “skeletal dysplasia”, and “unexplained paediatric onset end stage renal disease”. However, it is not on the “tubulopathies” panel, which is perhaps the most appropriate panel selection. For most cases where GEL missed the diagnosis, the panel selected was not inappropriate per se, but did not encompass the exact panel required, again highlighting why ‘agnostic to phenotype’ approaches are critical to ensure increased diagnostic yield, particularly in the UK where gene panels are the selected analytical method.

In view of the recognition of diagnoses outside of the selected gene panel(s), some NHS accredited laboratories adopted a policy, when reviewing results from the 100KGP, to assess de novo variation and Exomiser top-ranked results to uplift the resultant diagnoses. This approach to reporting has been informative for the strategy within subsequent large-scale WGS endeavors. When including these results, 26% of all causal variants returned by NHS labs in the 100KGP were not in the initial gene panel applied, exemplifying the issue with gene panel analysis strategies (Rehm 2022). However, re-analyses involve re-visiting sequencing data and clinical cases, something which could be mitigated by screening and prioritizing highly putative diagnostic variants in the first instance. Using DeNovoLOEUF as a screening strategy would have immediately identified 321 pathogenic variants, saving considerable time and money. On average, DeNovoLOEUF added only one extra variant for assessment in ~ 3% of all rare disease probands (0.023 variants per person).

Our method is rapid, having identified 172 variants in 2019 that we were unable to efficiently return to the patient’s clinical teams as the processes for returning research results to the clinic were not supported at scale. As a result of our collaboration with colleagues at Genomics England, a form that enables the submission of multiple potential diagnoses for different participants via a single submission within the RE is now in place. DeNovoLOEUF utilizes an effective screening approach to detect highly penetrant putative diagnostic variants across a large cohort. It should however be noted that whilst 285/333 (86%) of all the variants identified were fully diagnostic, 40/333 (12%) were a partial diagnosis, meaning that the variant was considered pathogenic but did not fully explain the phenotype. Additionally, following manual curation, there were ten variants whereby we were unable to confirm whether the variant explained the phenotype; all these patients lacked sufficient clinical data to determine causality, even though five of the variants were pathogenic by ACMG-AMP guidelines. These patients had a median of 4 HPO terms compared with 18 HPO terms for patients with a diagnosis. This highlights some of the challenges with phenotyping in a large-scale national sequencing project and that using HPO terms are sometimes insufficient to make a diagnosis and post-analysis communication with the clinical care team is a critical component of molecular diagnosis as emphasized by ACMG clinical practice guidelines (Bush et al. 2018). Genomics England is actively supporting improvements at the clinical–research interface to enable collaborations between researchers and clinicians and in patient phenotyping by the provision of Hospital Episode Statistics data within the RE as a longitudinal record of participants’ phenotypes.

With ever-increasing application of genome sequencing and a drive to sequence a further 5 million genomes in the UK, there is clear demand to find efficient analytic strategies. We attest our method to be a suitable adjunct to the current protocols to identify causal variation in 100KGP, the NHS Genomics Medicine Service, or other similar international initiatives. DeNovoLOEUF is capable of prioritizing putative pathogenic variants for diagnostic laboratories, saving time, and resources. Furthermore, one of the drawbacks of applying gene panels is that many are already outdated at the point of use, with new genes being consistently added to the literature. Our method can be easily applied iteratively for re-analysis as new genes are discovered and added to ClinVar (Landrum et al. 2014), HGMDPro, or GenCC (DiStefano et al. 2022), before being indexed in OMIM. However, at the time of method development, OMIM represented the best available repository of disease genes with a standardized method for curating genotype–phenotype relationships. We did however re-run DeNovoLOEUF in 2022 on new disease genes (added to GenCC) after our initial analysis in 2019; this identified a further 13 variants of which 12 have been confirmed as diagnostic.

Whilst the DeNovoLOEUF method has a high positive predictive value, 9 variants (1 from the 2021 analysis and 8 from curation analysis) were discordant with the molecular diagnosis returned. One variant was in an X-linked recessive gene and the patient was female. A further variant passed quality control filtering but was artefactual when the reads were directly visualised. Four variants were in a disease gene inconsistent with the patient’s phenotype and there was an average of 3 HPO terms per patient. One variant was within 6 base-pairs of a full splice rescue. Two variants were pLoF on a non-canonical transcript that was poorly expressed across disease-relevant tissues using the pext score (Cummings et al. 2019) based on GTeX data and intronic on the MANE Select transcript. Whilst one option would be to limit our method to variants on the MANE Select and MANE Plus Clinical transcripts, this is potentially problematic as not all genes have been curated to define additional transcripts to be included in the MANE Plus Clinical resource (Morales et al. 2022).

### Limitations and opportunities

Whilst our genotype-first method diagnosed patients missed by the initial 100KGP diagnostic strategy, it does not replace the importance of a phenotype-driven approach. It would be foolhardy not to look at all variants in a gene with a close phenotype match to the patient. Our method is best applied as a complementary screening strategy and will not diagnose the majority of patients, especially those with variants in non-constrained genes, or with pathogenic missense or extended splice site variants or with inherited variants. It also does not negate the need for variant curation, since some pLoF variants may not result in LoF and there is yet to be an automated method capable of replicating full manual curation (Gudmundsson et al. 2021). Furthermore, not all pLoF variants may be disease-causing through a loss-of-function mechanism, however we specifically selected genes constrained for LoF meaning we were enriched for pathogenic LoF variants in this specific subset of genes.

Our screening tool selects de novo variants, meaning that we excluded potential pathogenic variants in patients without trio data and excluded diseases where we may expect disease segregation, e.g., cardiac or immune disease. When applying the same filtering strategy, minus the de novo filter, to complex family structures or singletons, we identified an additional 270 diagnoses. This yielded a PPV of 36% vs 98% for trios. Used prospectively, this means that there is an increased probability of type 1 errors, although some of the unsubstantiated variants may represent real diagnoses. Unsurprisingly, for non-trio cases where returned diagnoses were discordant with our method, the median family size was 1, with 631/678 (93%) of cases being singletons.

We selected genes in the first LOEUF decile to increase the specificity of our method. As shown in the flagship gnomAD paper, a LOEUF score of < 0.2 is the most highly enriched for haploinsufficient disease genes. In total across all genes, there were 2074 de novo pLoF variants called in Genomics England of which 480/2074 (23%) were diagnoses. Of these, 79.2% were in LOEUF constrained genes (score < 0.2). Expanding our approach to de novo pLoF variants in disease genes using a higher LOEUF threshold will inevitably increase the diagnostic yield; however, this must be balanced with increased noise, an increased number of variants for review, and significantly reduced specificity. When expanding the analysis to a LOEUF score between 0.2 and 0.4, the positive predictive value reduced to 69%, and with a LOEUF score between 0.4 and 0.6, this reduced again to 45%. We recommend that this approach could be adopted for downstream analyses.

DeNovoLOEUF also risks identifying incidental findings including those in the ACMG secondary findings v3 list, which are a consequence of WGS and a tradeoff for increased diagnostic yield. Applying a LOEUF cut off < 0.2 identified 10 genes in the ACMG list, although these genes could be manually removed from DeNovoLOEUF if preferred, or if the patient has not consented for secondary findings (Miller et al. 2021).

**Fig. 5:**
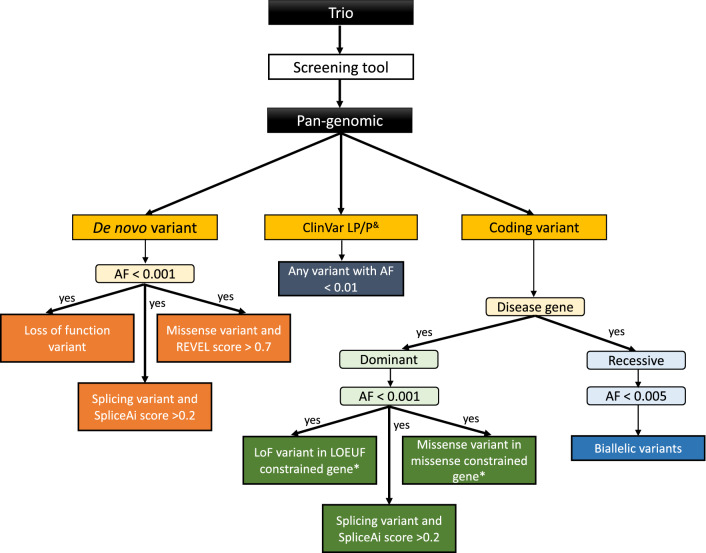
Proposed genotype-first screening strategy. All de novo variants with an allele frequency (AF) < 0.001 would be filtered, and loss-of-function variants would be prioritized, in addition to missense variants with a REVEL score > 0.7, and splicing variants with a SpliceAI score > 0.2. All known ClinVar likely pathogenic (LP) and pathogenic (P) variants would be reviewed independent of zygosity and prioritized with an AF < 0.01. Novel coding variants (absent from population databases) in a known OMIM disease gene would be extracted and subdivided by disease mechanism. For dominant genes, variants are filtered with an AF of < 0.001. Retained variants would be prioritized if they were a loss-of-function (LoF) variant in a LOEUF constrained gene; a missense variant in a missense constrained gene, using a z-score > 3 using the statistical method described by Samocha et al. (Samocha et al. 2014); or a splice site variant with a SpliceAI score > 0.2. For recessive genes, variants are filtered with an AF < 0.005. Any biallelic (phased) variants are retained. *Denotes user-specified cut offs for LOEUF and z-score missense constraint which can be tailored (or removed) as required. ^&^Suggest using ClinVar 2* and above

Whilst DeNovoLOEUF has a high PPV, there are opportunities to refine our method to increase sensitivity at the expense of specificity. We propose a revised screening pipeline that will not only identify de novo variants in LOEUF constrained genes, but also screen for all known rare ClinVar pathogenic or likely pathogenic variants regardless of the gene panel applied (Fig. 5). Our suggestion is to place de novo variants, ClinVar variants and novel coding variants into a new tier for assessment by NHS clinical laboratories to complement the current tiering system (The 100,000 Genomes Project Pilot Investigators 2021). Adding this additional review approach, along with a phenotype-driven variant analysis, is consistent with recently released best practices in genome analysis released from the Medical Genome Initiative (Austin-Tse et al. 2022). From reviewing 20 genomes, we estimate that this approach would yield 3–9 potential additional variants per trio, using a LOEUF cut off < 0.35, a missense constraint z-score > 3 (Samocha et al. 2014), and likely pathogenic/pathogenic ClinVar entries with 2 stars or above. Our hope is to achieve a higher diagnostic yield per number of variants assessed by diagnostic labs, whereby we prioritize the most salient variants first.

## Conclusion

We present a targeted screening tool, DeNovoLOEUF, that can be applied at scale to rapidly identify putative pathogenic variants with a 98% positive predictive value. Our method complements current family-based analyses and can add value by identifying diagnostic variants missed by filtering strategies that adopt predefined disease-targeted gene panels. We have identified 39 pathogenic variants missed by the initial 100KGP variant prioritization strategy. With 5 million more genomes being sequenced on the NHS, and many other international sequencing studies underway, we believe that our method alongside the new GEL initiative to report on Exomiser top-ranked variants can help rapidly and effectively improve diagnostic efficiency and uplift diagnostic rates for the benefit of rare disease patients and their families.

## Supplementary Information

Below is the link to the electronic supplementary material.

Supplementary file1 Supplementary Data 1: Summary of high-level phenotypes in 100,000 Genomes Project (XLSX 20 KB)

Supplementary file2 Supplementary Data 2: Curation of remaining variants in NHS accredited laboratory (XLSX 26 KB)

Supplementary file3 Supplementary Data 3: Curation of 13 variants identified following iterative re-analysis of DeNovoLOEUF on new disease genes (XLSX 16 KB)

Supplementary file4 Supplementary Data 4: List of genes and their corresponding LOEUF score (TSV 607 KB)

## Data Availability

Access to the 100KGP dataset analysed in this study is only available as a registered GeCIP member in the Genomics England Research Environment, but restrictions apply to the availability of these data due to data protection and are not publicly available. Information regarding how to apply for data access is available at the following URL: https://www.genomicsengland.co.uk/about-gecip/for-gecip-members/data-and-data-access/. All data shared in this manuscript were approved for export by Genomics England. The datasets and code supporting the current study can be accessed within the Genomics England Research Environment. DeNovoLOEUF is available here: https://github.com/lecb/DeNovoLOEUF.
